# 3D‐reconstructions of zygospores in *Zygnema vaginatum* (Charophyta) reveal details of cell wall formation, suggesting adaptations to extreme habitats

**DOI:** 10.1111/ppl.13988

**Published:** 2023-08-23

**Authors:** Charlotte Permann, Martina Pichrtová, Tereza Šoljaková, Klaus Herburger, Pierre‐Henri Jouneau, Clarisse Uwizeye, Denis Falconet, Eric Marechal, Andreas Holzinger

**Affiliations:** ^1^ Department of Botany University of Innsbruck Innsbruck Austria; ^2^ Department of Botany, Faculty of Science Charles University Prague Czech Republic; ^3^ Institute of Biological Sciences, University of Rostock Rostock Germany; ^4^ Laboratoire Modélisation et Exploration des Matériaux IRIG, CEA, Univ. Grenoble Alpes Grenoble France; ^5^ Laboratoire de Physiologie Cellulaire et Végétale CEA, CNRS, INRAE, Univ. Grenoble Alpes Grenoble France

## Abstract

The streptophyte green algal class Zygnematophyceae is the immediate sister lineage to land plants. Their special form of sexual reproduction via conjugation might have played a key role during terrestrialization. Thus, studying Zygnematophyceae and conjugation is crucial for understanding the conquest of land. Moreover, sexual reproduction features are important for species determination. We present a phylogenetic analysis of a field‐sampled *Zygnema* strain and analyze its conjugation process and zygospore morphology, both at the micro‐ and nanoscale, including 3D‐reconstructions of the zygospore architecture. Vegetative filament size (26.18 ± 1.07 μm) and reproductive features allowed morphological determination of *Zygnema vaginatum*, which was combined with molecular analyses based on *rbc*L sequencing. Transmission electron microscopy (TEM) depicted a thin cell wall in young zygospores, while mature cells exhibited a tripartite wall, including a massive and sculptured mesospore. During development, cytological reorganizations were visualized by focused ion beam scanning electron microscopy (FIB‐SEM). Pyrenoids were reorganized, and the gyroid cubic central thylakoid membranes, as well as the surrounding starch granules, degraded (starch granule volume: 3.58 ± 2.35 μm^3^ in young cells; 0.68 ± 0.74 μm^3^ at an intermediate stage of zygospore maturation). Additionally, lipid droplets (LDs) changed drastically in shape and abundance during zygospore maturation (LD/cell volume: 11.77% in young cells; 8.79% in intermediate cells, 19.45% in old cells). In summary, we provide the first TEM images and 3D‐reconstructions of *Zygnema* zygospores, giving insights into the physiological processes involved in their maturation. These observations help to understand mechanisms that facilitated the transition from water to land in Zygnematophyceae.

## 1 INTRODUCTION

The conquest of land by plants is one of the most significant evolutionary episodes in Earth's history. Terrestrialization took place 470–450 MYA (million years ago; Ordovician period), based on the adaptation of streptophyte green algae to harsh aerial conditions and was seminal for the subsequent evolution of land plants (Embryophyta) (reviewed in Becker & Marin, 2009; Sanderson et al., 2004). Embryophyta and streptophyte algae form the phylum Streptophyta and, together with the residual green algae (Chlorophyta), account for the majority of Viridiplantae clade (excluding Prasinodermophyta) of Archaeplastida (Becker & Marin, 2009; Li et al., 2020). Streptophyte green algae are grouped into two major clades, the higher branching ZCC‐grade (Zygnematophyceae, Coleochaetophyceae, and Charophyceae) and the lower branching KCM‐grade (Klebsormidiophyceae, Chlorokybophyceae, and Mesostigmatophyceae) (de Vries et al., 2016). While it was long hypothesized that Charophyceae, an algal class, which exhibits a complex and structured body plan, was the sister group to land plants, it is now well established that Zygnematophyceae are the closest extant relatives to Embryophyta (de Vries et al., 2018; Leebens‐Mack et al., 2019; Wodniok et al., 2011; Zhong et al., 2014).

Zygnematophyceae, or as they were earlier termed—conjugating green algae, are widespread and live in different freshwater to semi‐terrestrial habitats (Hall et al., 2008). They are a morphologically diverse and species‐rich lineage for which a five‐ordered system has been suggested (Hess et al., 2022). The first genomes (*Closterium* sp., *Mesotaenium endlicherianum*, *Penium margaritaceum*, *Spirogloea muscicola*, *Zygnema circumcariantum* and *Zygnema cylindricum*) have just recently been published (Cheng et al., 2019; Feng et al., 2023; Jiao et al., 2020; Sekimoto et al., 2023). The eponymous genus *Zygnema* C. Agardh comprises more than 150 different described species, sharing two characteristic star‐shaped chloroplasts (Guiry, 2023; Kadlubowska, 1984; Kim et al., 2012; Novis, 2004; Randhawa, 1959; Rundina, 1998; Stancheva et al., 2012; Zarina et al., 2006). In polar hydro‐terrestrial environments, these algae are a dominant eukaryotic component of microbial mats and one of the main primary producers as they are well adapted to harsh polar climatic conditions (Elster, 2002; Pichrtová et al., 2018; Pichrtová, Hájek, & Elster, 2016). As members of *Zygnema* occur worldwide in a plethora of different environments, they also withstand abiotic strains like osmotic stress (Kaplan et al., 2013; Pichrtová, Hájek, & Elster, 2014), freezing (Hawes, 1990; Trumhova et al., 2019), enhanced UV radiation (Holzinger et al., 2009; Holzinger et al., 2018) and desiccation (Pichrtová, Kulichová, & Holzinger, 2014). Such abilities are required for their persistence in semi‐terrestrial habitats as those abiotic stresses increase during the transition from water to land. In the vegetative state, their adaptation strategies involve the accumulation of cellular storage compounds and protective compounds as well as the thickening of the cell wall (Herburger et al., 2015, 2019; Holzinger et al., 2018; Pichrtová, Arc, et al., 2016). There are also different types of asexually formed resting stages, like (pre‐) akinetes, aplanospores or parthenospores, enabling them to tolerate unfavorable environmental conditions (Fuller, 2013; Kadlubowska, 1984). Besides the remarkable adaptation abilities of vegetative cells, sexual reproduction in Zygnematophyceae is considered another crucial yet underexplored factor in the process of terrestrialization.

Zygnematophyceae reproduce sexually by conjugation. Conjugation involves the transformation of vegetative cells into gametangia and the protoplasts into gametes, which fuse via a conjugation tube (Kadlubowska, 1984). Gametes lack organelles for locomotion, like flagella, and conjugation usually takes place in moist environments, however, it is not directly dependent on the availability of water, which might be another beneficial factor in semi‐terrestrial environments. Conjugation can occur in a variety of modes, categorized into three main groups. Scalariform conjugation involves the formation of a conjugation tube between cells of two different filaments, giving a ladder‐like appearance. If two neighboring cells from the same filament conjugate, it is termed lateral or terminal, the distinction being made by the origin of the conjugation tube (Kadlubowska, 1984). Further subdivisions are based on the location of gamete fusion (extra‐, bi‐, and monogametangial). The fused gametes develop into zygospores, which are diverse in form, color and structure. Classical zygnematophycean taxonomy is heavily based on the features of the conjugation process and the zygospores (Stancheva et al., 2012), as the simple morphology of the vegetative filaments is only informative on the genus level. A phylogenetic study on Arctic *Zygnema* populations highlighted an unsuspected hidden diversity within communities composed of morphologically very similar filaments, stressing the importance of a full life cycle for species determination (Pichrtová et al., 2018). Unfortunately, the induction of sexual reproduction under laboratory conditions has only been successful in a few cases in *Zygnema* and other genera (Czurda, 1930; El‐Sheekh et al., 2018; Ikegaya et al., 2012; Permann, Herburger, Felhofer, et al., 2021; Pfeifer et al., 2022; Renkert, 1987; Takano et al., 2019; Zwirn et al., 2013). The majority of the successful studies were conducted on *Spirogyra* sp. and suggest the need for higher light intensities and nitrogen depletion as important external triggers (El‐Sheekh et al., 2018; Permann, Herburger, Felhofer, et al., 2021; Takano et al., 2019). The lack of sexually reproducing cultures of *Zygnema* limits the research on the conjugation morphology of Zygnematophyceae and their zygospores and, ultimately, the study of plant terrestrialization.

In *Zygnema*, studies describing sexual reproduction and zygospore morphology are limited as most cultures are sterile. Czurda (1930) induced conjugation in species like *Zygnema circumcarinatum*, for which he assigned a heterothallic mating nature. While for this species, conjugation has been experimentally induced successfully in the laboratory (Gauche, 1966; Miller, 1973; Renkert, 1987), no recent reports of conjugating cultures are available, which might also be related to the fact, that the respective mating type (SAG 698‐1a) was lost and the strain kept under this accession number in the culture collection of algae in Göttingen (SAG) is most likely *Z. cylindricum* (Feng et al., 2021). Nevertheless, studies on field‐sampled material provide further insights. One sexually reproducing sample of *Zygnema* was recorded by Novis (2004) from New Zealand, and zygospores from three different species were reported by Poulícková et al. (2007) from the Czech Republic. However, in the latter case, the species determination was only successful for one sample, as a complete lifecycle was not observed in the others. More recently, phylogenetic analyses and light microscopical descriptions of the conjugation morphology have been published for *Zygnema* strains from California (Stancheva et al., 2012) and South Korea (Kim et al., 2012). Such studies, linking morphological species assignments to genetic data, are crucial for unraveling the phylogenetic relationships within *Zygnema*. However, to the best of our knowledge, no transmission electron microscopic (TEM) analysis or 3D‐reconstructions of the formed zygospores have been conducted in the genus *Zygnema* so far. This is a major gap in knowledge, as the zygospore cell wall structure and the internal cell architecture might provide crucial information on zygospore maturation mechanisms and their suspected high resistance to stress.

Zygospores of Zygnematophyceae are unique in their cell wall composition and architecture, differing greatly from their vegetative counterparts. The zygospore cell wall is much more complex and composed of three main layers, termed endo‐, meso‐, and exospore (Kadlubowska, 1984). The inner (endospore) and outer layer (exospore) are both colorless and contain mainly polysaccharides (Permann, Gierlinger, & Holzinger, 2022; Permann, Herburger, Felhofer, et al., 2021; Permann, Herburger, Niedermeier, et al., 2021; Poulícková et al., 2007). In *Spirogyra* and *Zygnemopsis* zygospores, an internal helicoidal order of the microfibrils was observed in these two layers (Permann, Gierlinger, & Holzinger, 2022; Pichrtová et al., 2018). Helicoidal patterns give the cell wall a range of different levels of viscoelasticity and stiffness (Roland et al., 1987). Such properties might be important for the development and maturation of resistant cells, like zygospores. The central mesospore is the surface structure‐ and color‐defining part of the zygospore, making it an important criterion for the infrageneric classification of *Zygnema* (Stancheva et al., 2012). This layer is composed of lipids and aromatic compounds (Permann, Gierlinger, & Holzinger, 2022; Permann, Herburger, Felhofer, et al., 2021; Permann, Herburger, Niedermeier, et al., 2021; Poulícková et al., 2007). In their spectral signature, the aromatics resemble those found in *Lycopodium* spores and are composed of a resistant sporopollenin‐like material (Permann, Gierlinger, & Holzinger, 2022; Permann, Herburger, Felhofer, et al., 2021; Permann, Herburger, Niedermeier, et al., 2021; Poulícková et al., 2007). We hypothesize that (1) the presence of a multilayered zygospore cell wall with a complex internal structure and resistant components is beneficial for the high resilience of zygospores against abiotic stresses and (2) that the building blocks for this complex cell wall originate from storage compound rearrangements.

In the present study, we characterized a field‐sampled *Zygnema* population from Croatia phylogenetically by its *rbc*L sequence and analyzed the conjugation morphology, with emphasis on the development and architecture of the zygospore cell wall. We provide the first TEM images and 3D‐reconstructions by focused ion beam scanning electron microscopy (FIB‐SEM) of *Zygnema* zygospores of different maturation stages to follow the development. In light of their position as the immediate sister lineage to land plants, such information and the phylogenetic characterization of a *Zygnema* species will expand our understanding of this diverse genus and how streptophyte algae were able to adapt to land.

## 2 MATERIALS AND METHODS

### 2.1 Isolation and cultivation of the investigated strain

**FIGURE 1:**
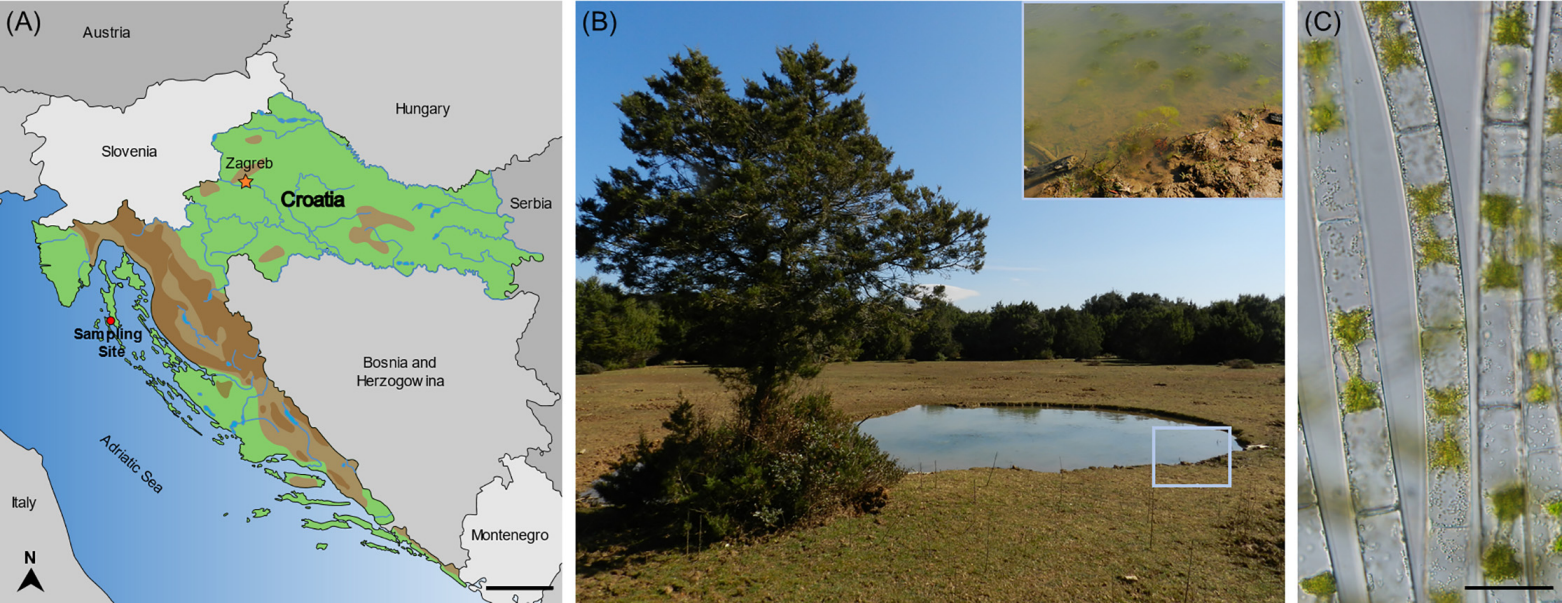
Sampling site and vegetative morphology of *Zygnema vaginatum*. (A) Map of Croatia, showing the sampling site at Osor. (B) Sampling site; inset: sampled algal mat. (C) Vegetative filaments of *Zygnema vaginatum*. Scale bars: (A) 50 km; (C) 50 μm.

Sampling was performed in March 2015 near the small town of Osor (Cres Island, Croatia; Figure 1A) in a shallow pool in the middle of a pasture, fully exposed to sunlight and most probably facing complete drought during summer (44°68′ N, 14°41′ O; Figure 1B). The pool contained floating patches of green algae (Figure 1B inset). Brief examination using a pocket microscope revealed vegetative filaments of *Zygnema* (Figure 1C) as well as conjugating stages. The pH (7.5) and the conductivity (450 μS cm^−1^) of the pool were measured on site using a WTW pH/Cond 340i device. Samples of biomass in water were kept in 80 mL plastic containers and transferred to Charles University (Prague, Czech Republic). A monoclonal culture was established and cultivated in Bold's basal medium (BBM; Merck). The culture was kept at 18°C and continuous illumination at 40 μmol m^−2^ s^−1^.

### 2.2 DNA isolation, PCR and phylogenetic analysis

DNA was isolated using the Instagene matrix (Bio‐Rad) as previously described (Ryšánek et al., 2015). The marker *rbc*L was used for the phylogenetic analysis, as it is the most commonly used gene in phylogenetic studies of Zygnematophyceae. The *rbc*L region was amplified using the primers RH1 and 1385R (McCourt et al., 2000). The PCR reaction was carried out with AmpliTaq GOLD 360 polymerase following the protocol described by Pichrtová et al. (2018). The PCR products were purified using AMPure XP beads (Beckman Coulter, Inc.) and sequenced by Macrogen Inc. Forward and reverse reads were assembled and edited using the SeqAssem program (Hepperle, 2004). The obtained sequence was submitted to GenBank under accession number OQ318179 under the name 5ChO1. A 1290 nucleotide long alignment was created in MEGA 7 (Kumar et al., 2016) using 23 additional *Zygnema* sp. sequences obtained from public databases to cover all major lineages of the genus (Pichrtová et al., 2018). Substitution model GTR + I + gamma was selected using MrModelTest 2.3 (Nylander, 2004) and the Akaike Information Criterion. The phylogenetic tree was inferred by Bayesian inference (BI) using MrBayes version 3.2.1 (Ronquist & Huelsenbeck, 2003). Two parallel Markov chain Monte Carlo runs were carried out for 5,000,000 generations, each with one cold and three heated chains. Trees and parameters were sampled every 100 generations, and trees from the initial 1000 generations were discarded using the sumt burnin function. Bootstrap analysis was performed by maximum likelihood (ML) in Garli 2.0 (Zwickl, 2006) and PAUP* Portable version 4.0 b10 (Swofford, 2002). The graphical adjustment of the final tree was performed using FigTree v1.4.4 (Rambaut, 2018) and Boxy SVG Editor (https://boxy-svg.com/). The alignment of the sequences is deposited as File (S1).

### 2.3 Light microscopy and toluidine blue staining

Light microscopical images of the zygospores were taken on a Zeiss Axiovert 200 M light microscope (Carl Zeiss AG), equipped with an Axiocam MRc5 or HRc camera (Carl Zeiss AG) controlled by Zeiss Axiovision software. Images of vegetative filaments and conjugating stages were taken on an Olympus BX61 (Olympus) microscope equipped with an Olympus DP73 camera. Semithin sections (~0.6 μm) of chemically fixed material (see below) were prepared with a Reichert Ultracut (Leica Microsystems), collected on glass slides, incubated with 0.3% toluidine blue for 5 min at 50°C and washed with distilled water.

### 2.4 Transmission electron microscopy

Chemical fixation of *Zygnema vaginatum* zygospores followed the protocol of Holzinger et al. (2009). In short, cells were fixed in 2.5% glutaraldehyde in 20 mM cacodylate (pH = 7), embedded in 3% agarose, post fixed in 1% OsO_4_ (in 20 mM cacodylate buffer) at 4°C overnight and dehydrated with ethanol and propylene oxide. Fixed samples were embedded in modified SPURR's resin, and ultrathin sections (~60 to 90 nm) were prepared using a Reichert Ultracut (Leica Microsystems). Sections were counterstained with 2% uranyl acetate and Reynold's lead citrate. Transmission electron micrographs were taken on a Zeiss Libra 120 transmission electron microscope (Carl Zeiss AG) at 80 kV, which was equipped with a 2 × 2k digital high speed or a TRS 2 k SSCCD camera and operated by ImageSP software (Albert Tröndle Restlichtverstärker Systeme).

### 2.5 Focused ion beam scanning electron microscopy and 3D image generation

Focused ion beam (FIB) tomography was performed with a Zeiss CrossBeam 550 microscope (Zeiss), equipped with Fibics Atlas 3D software for tomography as described previously (Uwizeye et al., 2021). The resin block containing the cells was fixed on a stub with silver paste and surface‐abraded with a diamond knife in a microtome to obtain a perfectly flat and clean surface. The entire sample was metallized with 4 nm of platinum to avoid charging during the observations. Inside the FIB‐SEM, a second platinum layer (1–2 μm) was deposited locally on the analyzed area to mitigate possible curtaining artifacts. The sample was then abraded slice by slice with the Ga+ ion beam (generally with a current of 700 nA at 30 kV). Each freshly exposed surface was imaged by scanning electron microscopy (SEM) at 1.5 kV and with a current of ~1 nA using the in‐column EsB backscatter detector (Movies S2–S4).

The process of image segmentation, 3D reconstruction and data visualization was performed using the image analysis software 3D Slicer following the previous workflow (Uwizeye et al., 2021). In summary, the stacks of FIB‐SEM images were registered, cropped and binned once in Fiji to be processed later and visualized in 3D Slicer.

## 3 RESULTS

### 3.1 Phylogenetic position of the newly isolated Croatian *Zygnema* strain

**FIGURE 2:**
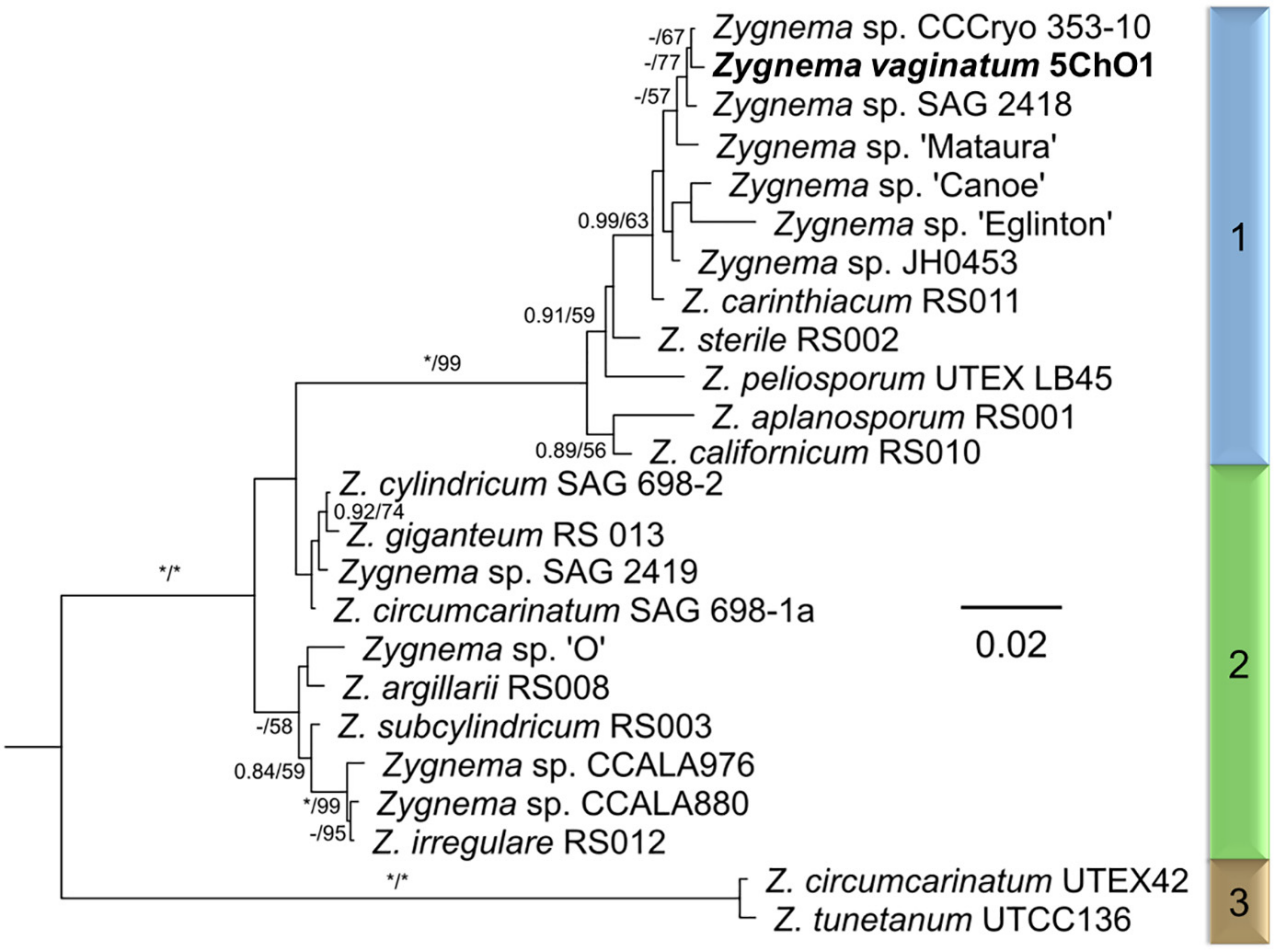
Phylogenetic tree of *Zygnema* sp. showing the position of *Zygnema vaginatum* in clade 1, as determined by Stancheva et al. (2012) and Pichrtová et al. (2018). A midpoint‐rooted BI of *rbc*L sequences is shown. Values at branches indicate Bayesian posterior probabilities (BI PP) and maximum likelihood (ML) bootstrap values (BS). Asterisks indicate BI PP = 1.00, and ML BS = 100; dashes indicate BI PP <0.8 and ML BS <50. Taxon labels include the NCBI/EMBL accession number and strain designation. The scale bar indicates the expected number of substitutions per site.

Analysis of the *rbc*L sequence placed the isolated *Zygnema* strain in clade 1, as determined by Stancheva et al. (2012) and Pichrtová et al. (2018) (Figure 2). It was closely related to *Zygnema* sp. “S” (CCCryo 353‐10) obtained from the south of Svalbard, but from this strain, no reproductive stages or zygospores were reported (Pichrtová et al., 2018). Two other closely related *Zygnema* strains were originally isolated from Austria (*Zygnema* sp. SAG 2418) and New Zealand (*Zygnema* sp. “Mataura” https://www.ncbi.nlm.nih.gov/nuccore/949395517, Novis et al. unpublished) and again, no reproductive stages were reported from these strains (Herburger et al., 2015).

### 3.2 Conjugation and zygospore morphology

**FIGURE 3:**
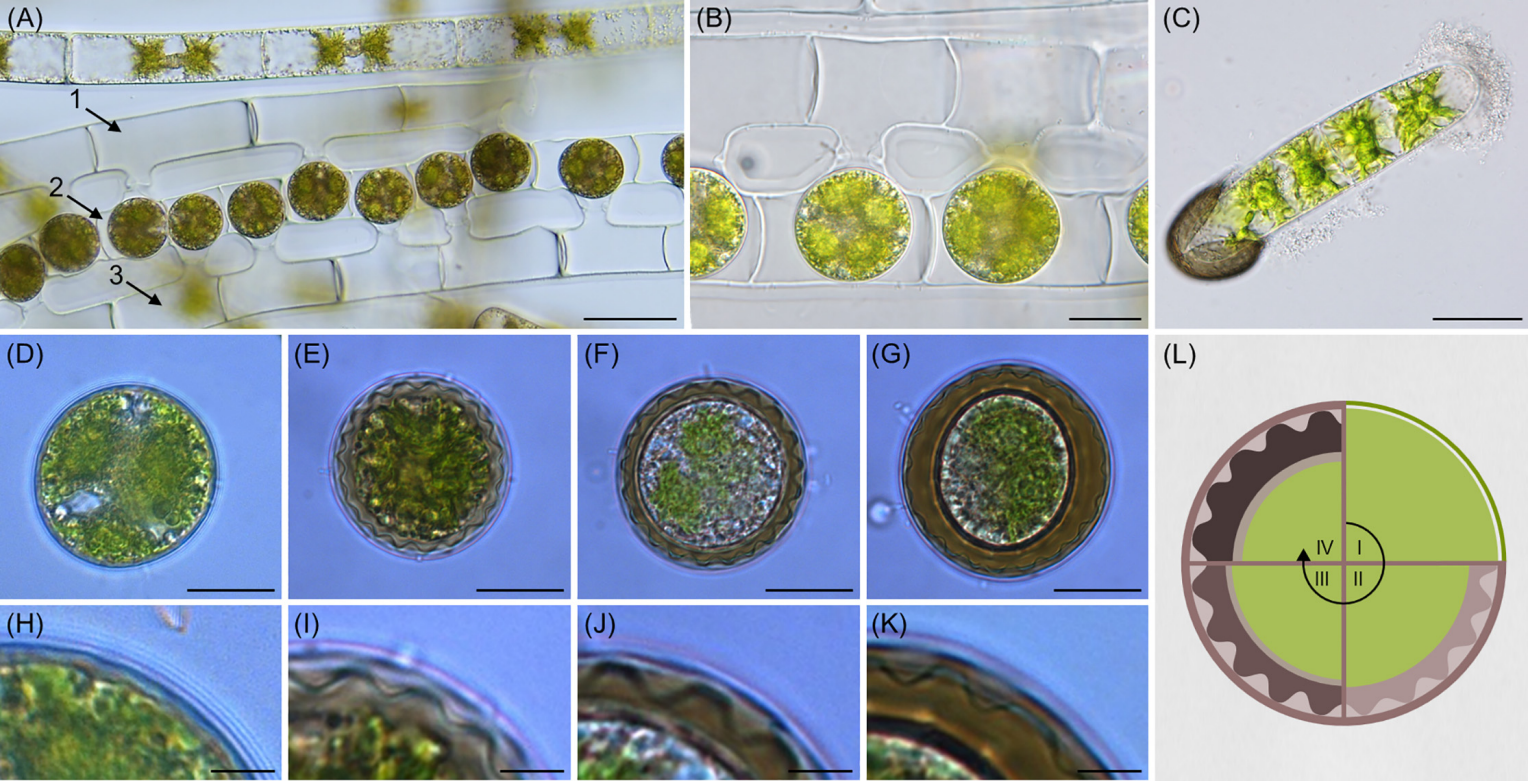
Conjugation, zygospore germination and maturation of *Zygnema vaginatum*. (A) Vegetative filament and monogametangial scalariform conjugation involving three different filaments (arrows). (B) Detail view of young zygospores formed by scalariform conjugation. (C) Germinating zygospore. (D–G) Young to mature zygospores with increasing cell wall thickness and (H, I) corresponding detail view of the cell wall: (D, H) Young zygospore; (E, I) Zygospore with structured light brown mesospore layer; (F, J) Zygospore with further thickened cell wall; (G, K) Mature zygospore with massive cell wall. (L) Schematic representation of the zygospore maturation (I–IV) with increasing cell wall thickness. Scale bars: (A) 50 μm; (B) 20 μm; (C) 40 μm; (D–G) 10 μm; (H–K) 2.5 μm.

**FIGURE 4:**
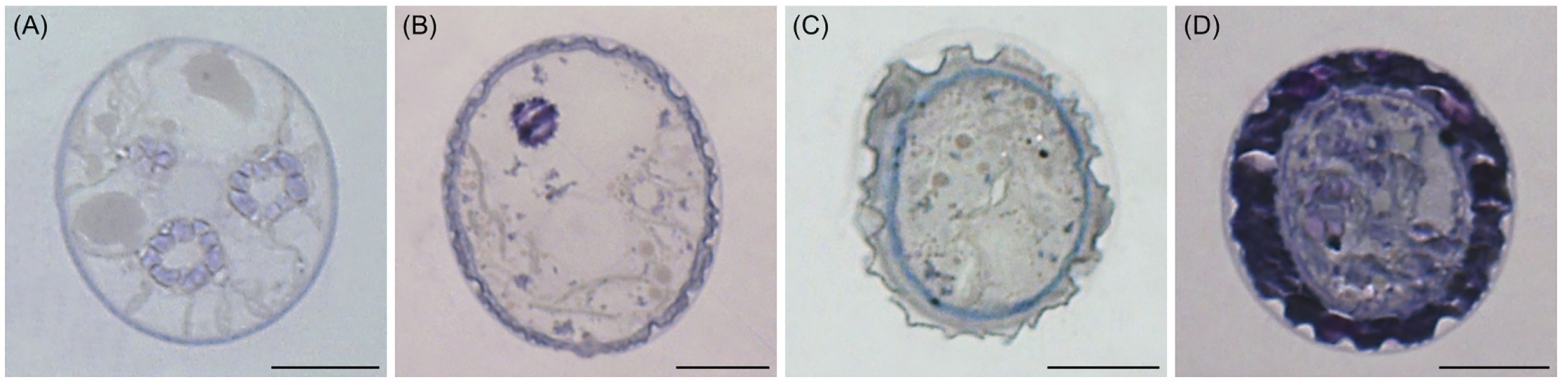
Semithin sections of *Zygnema vaginatum* zygospores stained with toluidine blue. (A) Young zygospore with thin cell wall. (B) Developing zygospore with increasing cell wall thickness. (C) Zygospore with developing cell wall. (D) Mature zygospore with massive cell wall. Scale bars: (A–D) 10 μm.

The vegetative filaments of the field‐sampled *Zygnema* sp. strain exhibited two genus‐specific lobed chloroplasts and a cell width of 26.18 ± 1.07 μm (*n* = 30). The conjugation was observed as monogametangial and scalariform (Figure 3A,B). The ladder‐like arrangement sometimes involved three different filaments, with the resulting zygospores forming in the gametangia of the middle filament (Figure 3A). The female gametangia, containing the zygospores, lacked swelling (Figure 3B). Germination of the new filament followed a split of the zygospore cell wall into equal halves (Figure 3C). Light microscopical images showed freshly formed zygospores, which exhibited a thin single‐layered cell wall similar to their vegetative counterpart (Figure 3D,H). During maturation, the zygospore wall developed into a multilayered thick cell wall (Figure 3E–G,I–K). The middle layer (mesospore) was brown and exhibited distinct semi‐circular indentations (Figure 3E–G). Different maturation stages (I‐IV) are illustrated in Figure 3L. Semithin sections stained with toluidine blue showed young zygospores with a single‐layered cell wall (Figure 4A) and more mature cells, developing a complex tripartite cell wall with an extremely thick and sculptured mesospore (Figure 4B–D).

### 3.3 *Zygnema vaginatum* zygospore ultrastructure

**FIGURE 5:**
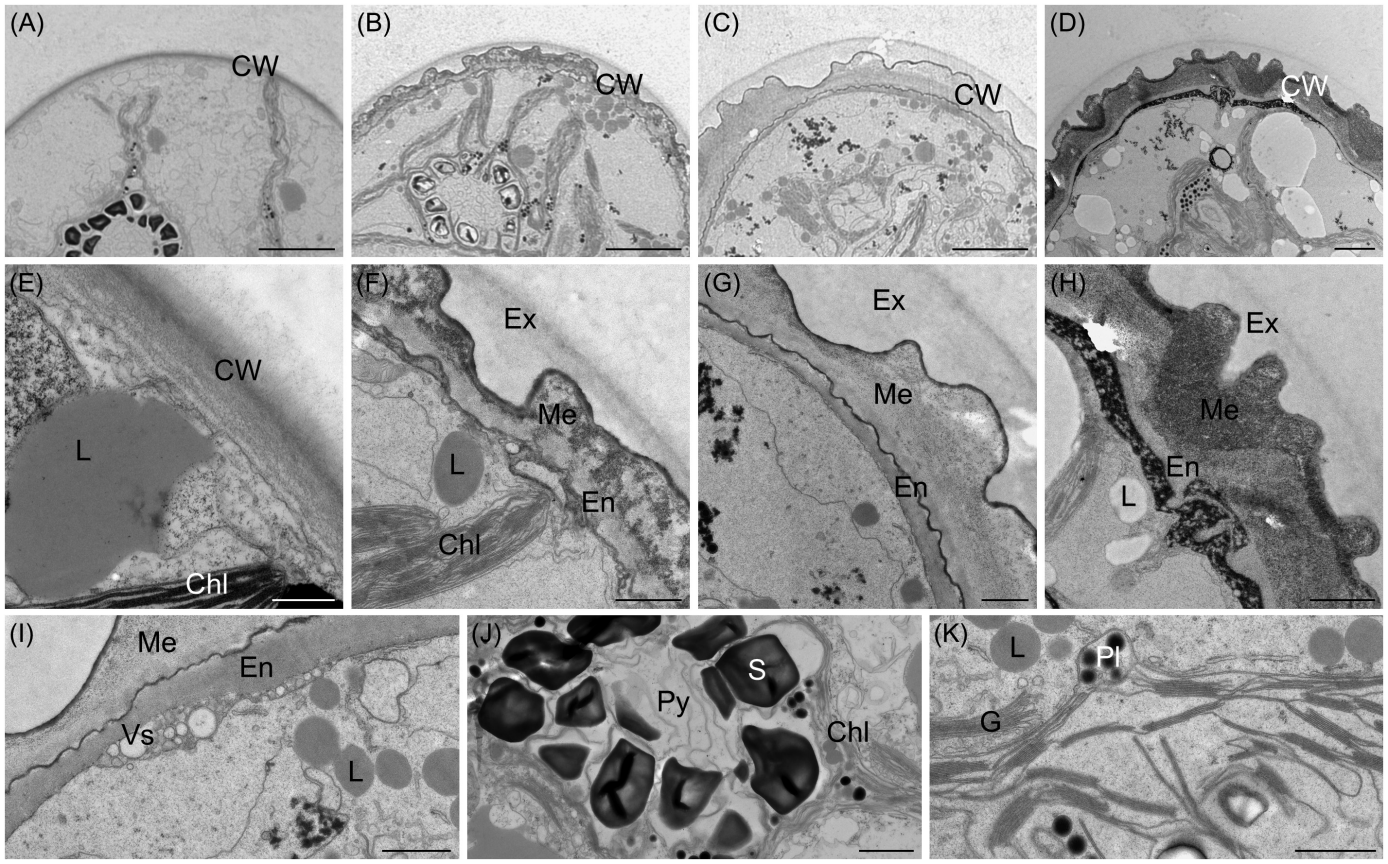
Transmission electron micrographs of *Zygnema vaginatum* zygospores. (A–D) Young to mature zygospores with increasing cell wall thickening and (E–H) corresponding detail view of the cell wall: (A) Young zygospore with thin cell wall; (B) Zygospore with developing cell wall; (C) Zygospore wall displaying a strongly sculptured mesospore; (D) Mature zygospore with thick multilayered cell wall; (E) Beginning zygospore cell wall development; (F) Differentiated zygospore cell wall; (G) Three‐layered zygospore cell wall; (H) Thick mature zygospore cell wall. (I) Vesicles accumulating at the endospore. (J) Pyrenoid surrounded by starch granules and chloroplast lobes. (K) Cell lumen with lipid droplets, plastoglobules and Golgi bodies. Chl, chloroplast lobe; CW, cell wall; En, endospore; Ex, exospore; G, Golgi body; L, lipid droplet; Me, mesospore; Py, pyrenoid; Pl, plastoglobules; S, starch granule; Vs, vesicle. Scale bars: (A–C) 5 μm; (D) 2 μm; (E) 500 nm; (F–K) 1 μm.

Ultrastructural investigations by TEM revealed that the zygospore wall became more complex during maturation. Young zygospores exhibited a thin cell wall, which resembled those of vegetative cells (Figure 5A). During maturation, the zygospore wall increased in thickness and complexity (Figure 5B–D). Initially, the polysaccharide‐rich exospore (outer layer) exhibited a loose multilayered architecture, which developed into a smooth layer covering the mesospore (Figure 5E–H). In contrast, the mesospore was highly sculptured with deep indentations on its surface. Facing the cell lumen, this middle layer was also sculptured, though less pronounced than described above (Figure 5F–H). The adjacent endospore was the thinnest layer, appeared to be rich in polysaccharides and was denser than observed for the exospore (Figure 5F–H). Vesicles accumulating beneath the endospore of a maturing zygospore were observed (Figure 5I). In the cell lumen, pyrenoids surrounded by starch granules and chloroplast lobes, as well as a high abundance of Golgi bodies were visible (Figure 5J,K). Additionally, lipid droplets (LDs) in varying shapes, sizes and electron densities were found in the cell lumen (Figure 5E,H).

### 3.4 Internal cell and cell wall architecture of developing zygospores

**FIGURE 6:**
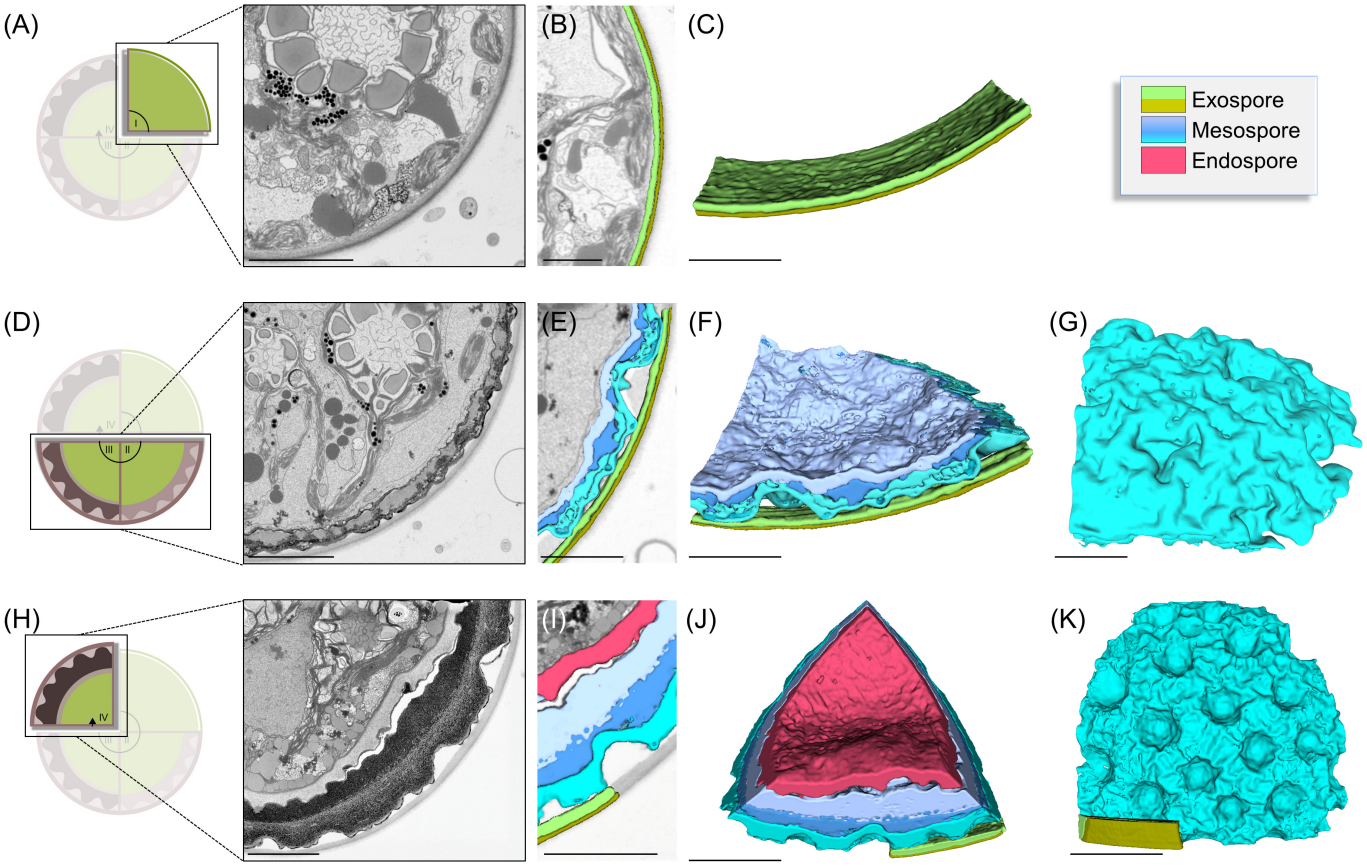
Cell wall architecture of *Zygnema vaginatum* zygospores by 3D‐reconstructions following FIB‐SEM. (A–C) Young developmental state. (D–G) Intermediate developmental state. (H–K) Mature developmental state. (A, D, H) Schematic representation of developmental state and section segmented from FIB‐SEM images (stack of frames in Movies S2–S4). (B, E, I) Section of the cell wall with (C, F, J) corresponding 3D‐reconstruction of the cell wall layers. (G, K) Surface structure of the cell wall. Scale bars: 5 μm.

We reconstructed the 3D‐architecture of developing zygospores, represented by three different maturation stages (young, intermediate and mature; Figure 3L), by FIB‐SEM imaging. The young zygospore (Figure 6A) exhibited only two thin wall layers with a smooth structure (Figure 6B,C). The intermediate developmental stage (Figure 6D) depicted a more complex and multilayered cell wall with a structured mesospore (Figure 6E,F). The cell wall surface was characterized by multiple indentations (Figure 6G). The complexity and number of layers further increased during the maturation process, resulting in a massive multilayered cell wall in mature zygospores (Figure 6H–J), which exhibited a distinct surface structure with regular and deep indentations (Figure 6K).

**FIGURE 7:**
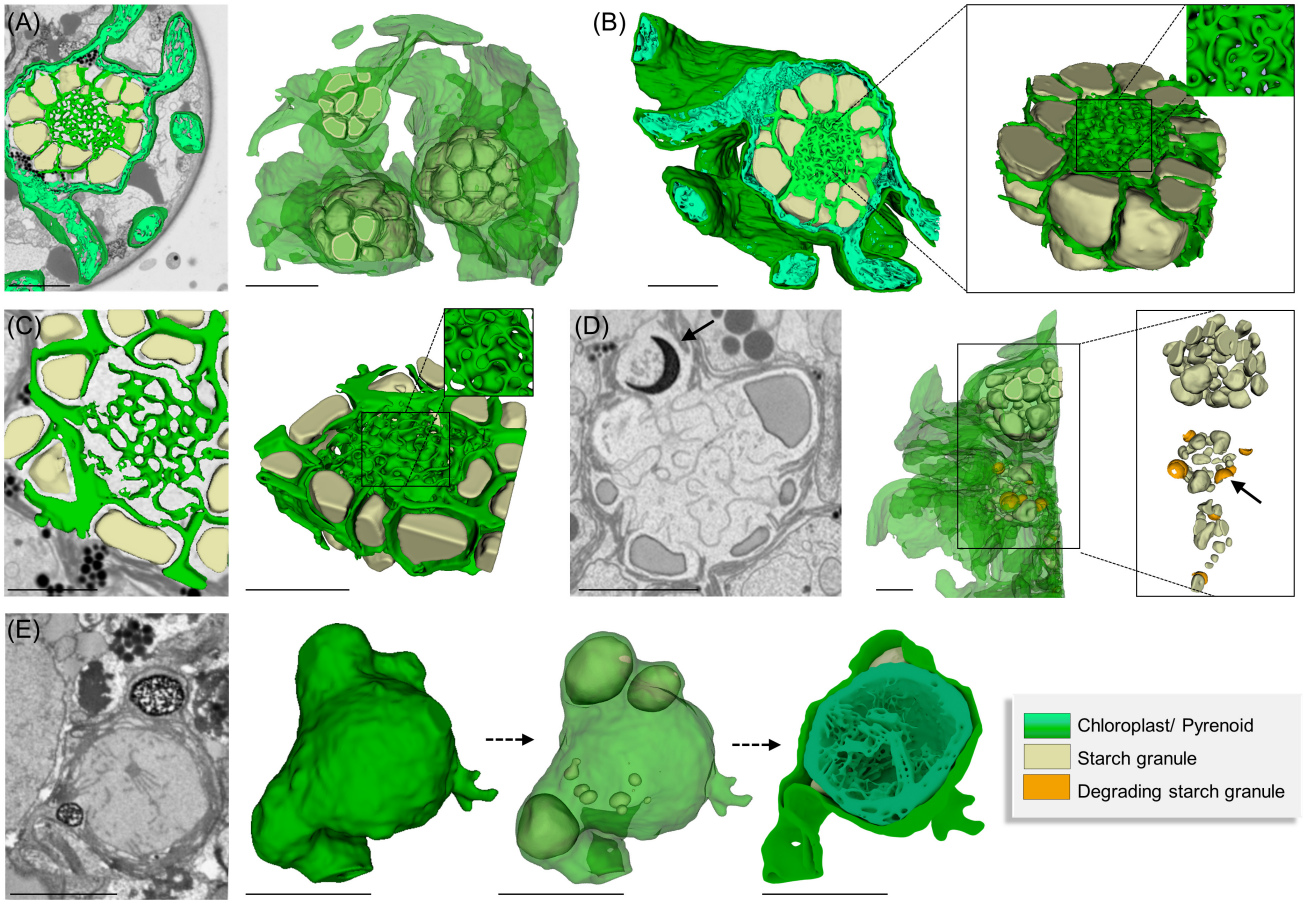
Chloroplast reorganization in *Zygnema vaginatum* zygospores during maturation illustrated by 3D‐reconstructions following FIB‐SEM. (A, B) Young developmental state. (C, D) Intermediate developmental state. (E) Mature developmental state. (A) Chloroplast and pyrenoids. (B) Detail view of a pyrenoid surrounded by starch granules. (C) Detail view of a pyrenoid surrounded by starch granules, the central part shows gyroid cubic membranes. (D) Chloroplast and pyrenoid with degrading starch granules (arrows). (E) Detail view of the chloroplast and a degrading pyrenoid. Scale bars: (A, B, D) 5 μm; (C, E) 2.5 μm.

**FIGURE 8:**
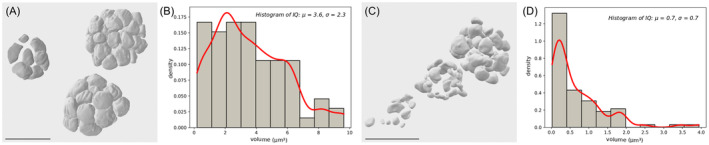
Starch distribution in *Zygnema vaginatum* zygospores. (A) 3D‐reconstructions of starch granules in young developmental stage with (B) corresponding distribution histogram. (C) 3D‐reconstructions of starch granules in intermediate developmental stage with (D) corresponding distribution histogram. Scale bars: 5 μm.

Regarding the reorganization of the internal structure, chloroplasts degraded during the maturation process. In young zygospores, the lobed chloroplasts and the pyrenoids were well developed and intact (Figure 7A,B), with the latter being surrounded by multiple starch granules and permeated by a dense network of intra‐pyrenoidal membranes in a gyroid‐like appearance (Zhan et al., 2017; Figure 7B). Reconstructions of the chloroplast and pyrenoid in an intermediate developmental stage revealed a decreased density of the intra‐pyrenoidal membranes as well as “crescent‐shaped” degrading starch granules (Figure 7C,D). Highly degraded pyrenoids surrounded by only a few starch granules and remnants of the intra‐pyrenoid membrane occurred in mature zygospores (Figure 7E). Comparisons of the starch granule size between the developmental stages revealed a mean volume of 3.58 ± 2.35 μm^3^ with a surface/volume ratio of 4.47 ± 2.11 (*n* = 70) in young cells (Figure 8A,B) and about 0.68 ± 0.74 μm^3^ and 8.06 ± 3.34 (*n* = 83), respectively, in intermediate cells (Figure 8C,D). In fully mature zygospores, the degradation level of starch granules had progressed too far for this analysis.

**FIGURE 9:**
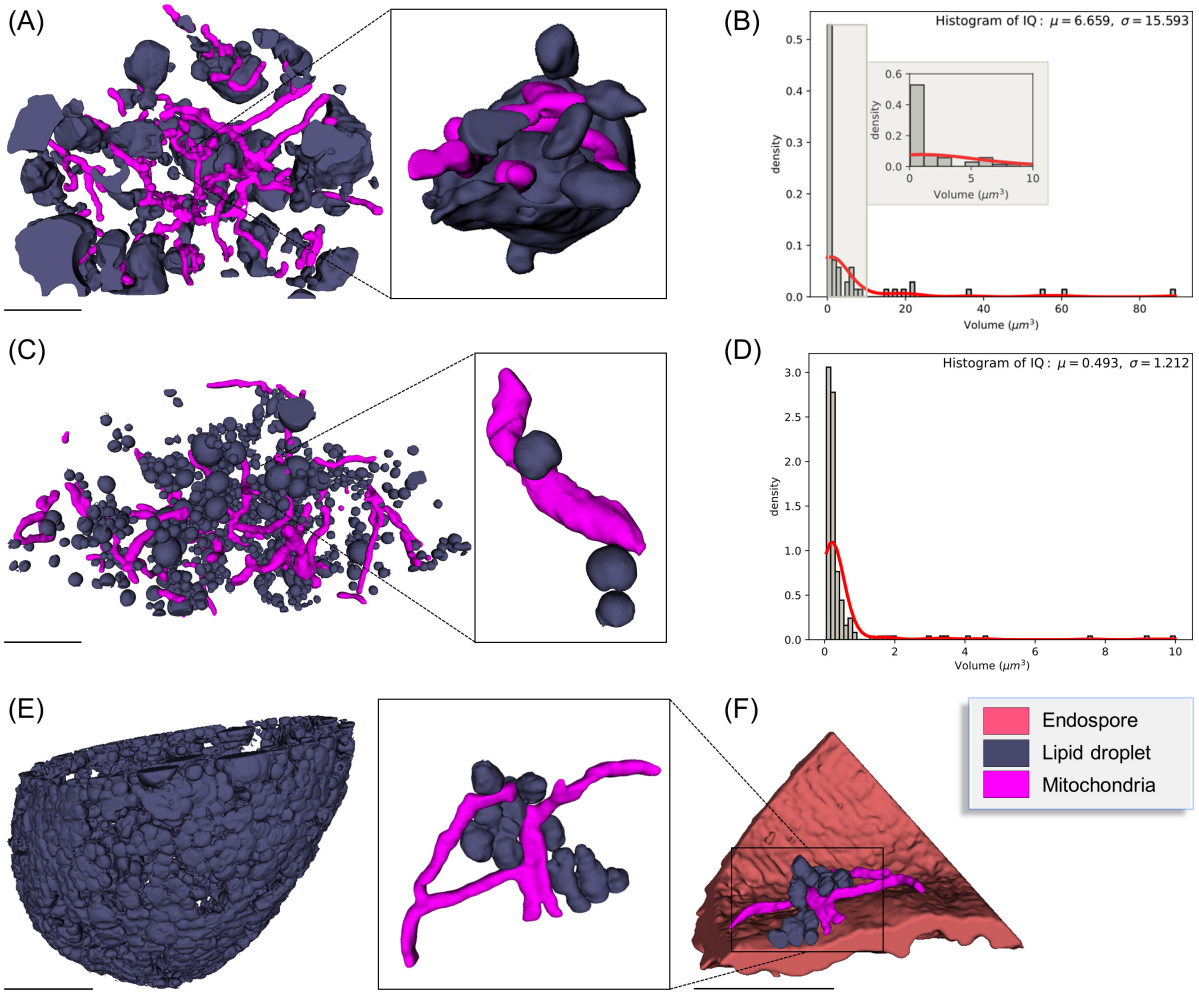
Lipid droplet reorganization in *Zygnema vaginatum* zygospores during maturation illustrated by 3D‐reconstructions following FIB‐SEM. (A, B) Young developmental state. (C, D) Intermediate developmental state. (E, F) Mature developmental state. (A, C) Lipid‐like bodies and mitochondria with (B, D) corresponding distribution histogram. (E) Lipid droplets. (F) Lipid droplets in direct contact with endospore and mitochondria. Scale bars: 5 μm.

In addition to the degradation processes of the chloroplasts and starch granules, a reorganization of LDs was reported during zygospore maturation. In the cell lumen of young zygospores, multiple LDs with a variety of shapes and sizes, with an average volume of 1.83 ± 3.03 μm^3^ (*n* = 63) and a surface/volume ratio of 7.23 ± 2.58 were detected (Figure 9A,B). Lipid droplets in the intermediate developmental stage were of spherical shape (average volume 0.49 ± 1.21 μm^3^; surface/volume ratio 9.04 ± 2.52) and sometimes found in direct contact with the innermost layer of the cell wall (Figure 9C,D). Analysis of the mature zygospores revealed a layer‐like distribution of LDs, which started to merge (Figure 9E). Similar as already observed in the intermediate state, they were found in direct contact with the cell wall (Figure 9F). LDs were also found in direct contact with network‐forming mitochondria in all developmental stages (Figure 9A,C,F), suggesting a possible metabolic utilization of fatty acids from triacylglycerol stored in lipid bodies via beta‐oxidation. A more detailed and quantitative analysis of the change of LDs during zygospore maturation showed that in young cells, approximately 11.77% of the total cell volume (calculated by fitting the shape with an ellipsoid, with the major axis and minor axis taken from the model mesh) were occupied by these lipid storage forms, while in the intermediate state, LDs accounted for 8.79% and for 19.45% in the mature zygospore.

## 4 DISCUSSION

In the present study, we characterized a newly isolated strain of *Zygnema* sp. from Croatia by phylogenetic analysis (*rbc*L sequence) paired with a morphological species determination based on vegetative and reproduction characteristics. We investigated the zygospore morphology by LM, TEM and FIB‐SEM. Observing a complete lifecycle in *Zygnema* (Zygnematophyceae) is rare, and our data provide an important foundation for more in‐depth studies on the zygospore wall biosynthesis, including the fundamental intracellular reorganization processes during spore development. Our study furthermore provides the first ultrastructural and 3D‐analysis of *Zygnema* zygospores.

### 4.1 Phylogenetic position and morphological description of the isolated *Zygnema* sp. strain

The isolated *Zygnema* strain, which was placed into clade 1 (clades defined by Pichrtová et al., 2018; Stancheva et al., 2012), exhibited a close relationship to strains isolated from Svalbard (*Zygnema* sp. S; CCCryo 353–10), Austria (*Zygnema* sp.; SAG2418) and New Zealand (*Zygnema* sp. “Mataura”) (Herburger et al., 2015; Pichrtová et al., 2018). Given that the investigated strain stemmed from Croatia, close phylogenetic relationships do not correlate with geographical distribution. In general, members of clades 1 and 2 have both been shown to exhibit a cosmopolitan distribution (Pichrtová et al., 2018; Stancheva et al., 2012). The vegetative cells of the investigated strain are 26.18 ± 1.07 μm wide (*n* = 30), resembling the size of genotypes *Zygnema* sp. P, V and M from Svalbard (Pichrtová et al., 2018). Interestingly, these three genotypes were phylogenetically assigned to clade 2. In contrast, the closely related *Zygnema* sp. S has a cell width of approximately 31.5 ± 4.5 μm (Pichrtová et al., 2018), and 18.65 ± 0.83 μm has been reported for *Zygnema* sp. SAG2418 (1–2 months old; Herburger et al., 2015). While the cell width of the vegetative filaments has been proposed to reflect their phylogenetic position (Herburger et al., 2015), the present study is in accordance with others that suggest that this trait has no phylogenetic relevance (Permann, Pierangelini, et al., 2022; Pichrtová et al., 2018), indicating a broad phenotypic plasticity in terms of cell width.

Using marker genes for resolving the phylogenetic relationships within the genus *Zygnema* is a widely used strategy, as often only sterile filaments can be obtained. However, traditional taxonomy and morphological species determination are based on the conjugation process and zygospore characteristics, such as the mesospore color (Kadlubowska, 1984; Stancheva et al., 2012). According to traditional morphological classification, the species investigated in the present study was assigned to the section Leiopsermum, where the zygospore locates in the female gametangium (Kadlubowska, 1984). The classification of *Zygnema* in different sections based on the presence/absence of sexual reproduction and the position of zygospores, however, does not reflect natural groups or correspond with the three clades assigned by molecular data (Stancheva et al., 2012). Within clades 1 and 2, the location of zygospore formation (in conjugation tube or in gametangia) nevertheless represents a distinct feature between the two groups (Stancheva et al., 2012). So far, *rbc*L sequences and associated descriptions of the zygospore morphology are available only for a few species of Zygnematophyceae (Stancheva et al., 2012). In that sense, phylogenetic analysis based on molecular data in conjunction with morphological descriptions, such as the present study, are vital in revising and completing the existing infrageneric classification of *Zygnema*.

Based on the conjugation characteristics, zygospore features and width of the vegetative filaments, the strain investigated in the present study was morphologically assigned as *Zygnema vaginatum* (first described by Klebs, 1886), according to Kadlubowska (1984). The cell widths of 25–27 μm stated in Klebs (1886) and 24–30 μm stated in Kadlubowska (1984) are in accordance with the observations of the present study (26.18 ± 1.07 μm). Concerning the reproductive features, the investigated strain exhibited a scalariform monogametangial conjugation, with no or only light swelling of the female gametangia. The zygospores were spherical, and the mesospore was thick and brown with circular indentations. These observed reproductive features of the conjugation and zygospore morphology also align with previous descriptions.

### 4.2 Maturing zygospores show a massive thickening of the cell, especially of the mesospore

Young zygospores were surrounded by a thin, colorless and smooth cell wall, and chloroplasts occupied most of the cell lumen. Maturing zygospore walls strongly increased in diameter and turned brown with a distinct surface structure. Earlier studies showed that the endo‐ and exospore are mainly composed of polysaccharide‐rich microfibrils with varying levels of complexity (Permann, Gierlinger, & Holzinger, 2022; Permann, Herburger, Felhofer, et al., 2021; Permann, Herburger, Niedermeier, et al., 2021; Pichrtová et al., 2018; Poulícková et al., 2007). While a helicoidal pattern was reported in zygospores of *Spirogyra* (Permann, Gierlinger, & Holzinger, 2022) and *Zygnemopsis* (Pichrtová et al., 2018), no indication for the occurrence of this arrangement was found in *Zygnema vaginatum*, investigated in the present study. Based on the chemical composition of the mesospore—as investigated in *Spirogyra* and *Mougeotia* by RAMAN spectroscopy—it is hypothesized that this layer acts as an effective protection against abiotic stresses like UV‐radiation and desiccation (Permann, Gierlinger, & Holzinger, 2022; Permann, Herburger, Felhofer, et al., 2021; Permann, Herburger, Niedermeier, et al., 2021). Protective features might be enhanced by lipid/aromatic and/or sporopollenin‐like compounds in the mesospore, which produce a similar spectral signature as found for *Lycopodium* spores (Permann, Gierlinger, & Holzinger, 2022; Permann, Herburger, Felhofer, et al., 2021; Permann, Herburger, Niedermeier, et al., 2021). The observed high electron density of this layer in the investigated *Zygnema* zygospores suggests a comparable chemical composition. The aromatic sporopollenin‐like material found in zygnematophycean zygospores has previously been suggested as “algaenans” (Blokker, 2000; Poulícková et al., 2007), which, however, are composed of nonhydrolyzable aliphatic biopolymers. Sporopollenin is synthesized by the phenylpropanoid pathway, while algaenan is formed via the acetate‐malate pathway (via lipid‐type compound synthesis; Poulícková et al., 2007). However, while the latter is common in Chlorophyta, Eustigmatophyta and Dinophyta, parts of the phenylpropanoid pathway, which has long been considered land‐plant specific, have been detected to play a major role in Zygnematophyceae (de Vries et al., 2021; Rieseberg et al., 2022; Serrano‐Pérez et al., 2022). As current data suggest that the phenylpropanoid pathway evolved within the lineage of Streptophyta and represents a key prerequisite for the process of terrestrialization, the aromatic compounds found in zygnematophycean zygospores by RAMAN spectroscopy (Permann, Gierlinger, & Holzinger, 2022; Permann, Herburger, Felhofer, et al., 2021; Permann, Herburger, Niedermeier, et al., 2021) most likely also originate from this pathway.

Concerning the ultrastructure of the zygospore wall, FIB‐SEM imaging analysis confirmed the intricate and multilayered structure of the cell wall, as suggested by LM and TEM investigations. The increase in layers and surface structure was ostensibly illustrated by the 3D‐reconstructions. Especially the middle layer, termed mesospore, was formed by three sublayers and contributed to a major part of the cell wall.

### 4.3 Chloroplast degradation and lipid droplet reorganization in maturing zygospores revealed by FIB‐SEM imaging

The analysis of three different developmental stages (young, intermediate, and mature) also allowed the documentation of changes and degradation processes of the chloroplast. After the fusion of the gametes, the male chloroplasts disintegrate, while the chloroplasts from the female gametangia persist in the developing zygospore (Kadlubowska, 1984). In zygotes of *Chlamydomonas*, it was shown that the Ezy‐1 polypeptide is involved in the selective degradation of the minus (mt^−^) chloroplast DNA, ensuring a uniparental inheritance of chloroplast traits (Armbrust et al., 1993). The degradation of the paternal cpDNA takes place almost immediately after zygote formation within the first few hours (Burton et al., 1979; Kuroiwa et al., 1982). Gradual degradation of pyrenoids and starch granules during the zygospore maturation was also observed in the present study. The intra‐pyrenoid membranes arranged in multilayered gyroid cubic shapes, as reported from vegetative cells (Zhan et al., 2017), decreased in density and were only present in fragments in the mature zygospores. The degradation of the male chloroplast and starch granules most likely provides energy and/or building blocks for the developing and consequently germinating zygospore. Chloroplast turnover is a known and vital part of many plant life cycles and includes a variety of different pathways (Zhuang & Jiang, 2019).

The LDs observed in the zygospores could indeed originate from the degradation of cell organelles, such as the male chloroplast(s). In pollen grains, the degradation of the tapetum, a tissue rich in lipidic compounds, is directly linked to the development of the pollen coat (Levesque‐Lemay et al., 2016; Parish & Li, 2010; Zienkiewicz & Zienkiewicz, 2020). As lipids have been suggested as components of the mesospore (Permann, Gierlinger, & Holzinger, 2022; Permann, Herburger, Felhofer, et al., 2021; Permann, Herburger, Niedermeier, et al., 2021), lipid reallocation from degrading paternal organelles might also play a vital role in the development of the zygospore wall. Studies on *Chlamydomonas reinhardtii*, inducing autophagy via chloroplast damage by inhibiting fatty acid (FA) synthesis, suggest links between lipid metabolism, chloroplast integrity, and autophagy (Heredia‐Martínez et al., 2018). While in zygospores of Zygnematophyceae, chloroplast degradation is triggered by gamete fusion rather than stress, increased autophagic degradation is an essential mechanism for providing building blocks, like amino acids and FAs, as well as energy sources. The recycling of whole organelles by autophagy provides precursors for triacylglycerol, loaded inside LDs (Rambold et al., 2015). Autophagosomes can also be involved in LD degradation via a process known as lipophagy (Singh et al., 2009; van Zutphen et al., 2014). Triacylglycerol contained in LDs can provide fatty acids for bioenergetic needs, via beta oxidation in the mitochondrion attached to its surface, or peroxisomes, as a longer‐term alternative carbon and energy resource to starch. The change in LD electron density, as observed by TEM, also suggests a changing FA composition or saturation level during their development (Cheng et al., 2009). In vegetative *Zygnema*, a drastic change in FA composition between young and pre‐akinete cells has been reported (Pichrtová, Arc, et al., 2016). Lipid droplets are not only efficient energy and carbon reservoirs but are also involved in a plethora of different membrane activities (Zienkiewicz & Zienkiewicz, 2020). Membrane trafficking components are required in their dynamics, that is, LD loading with triacylglycerol (TAG) synthesized in the endoplasmic reticulum, balanced with TAG hydrolysis by lipases driving fatty acids to the mitochondrion and/or peroxisome (Huang et al., 2019).

In seeds, the major structural proteins associated with LDs include oleosin, caleosin, and steroleosin (Zienkiewicz & Zienkiewicz, 2020). It is suggested that, during seed germination, LD degradation takes place inside protein bodies and that caleosin‐dependent macrolipophagy is involved in the regulation of LD turnover (Zienkiewicz et al., 2014; Zienkiewicz & Zienkiewicz, 2020). Putative genes encoding for caleosin‐like proteins have also been found in algae, nonvascular plants, and fungi (Jiang & Tzen, 2010; Rahman, Hassan, Hanano, et al., 2018; Rahman, Hassan, Rosli, et al., 2018; Zienkiewicz & Zienkiewicz, 2020). Moreover, an oleosin homolog was highly expressed in mature cells of *Zygnema circumcarinatum* (SAG 2419; Rippin et al., 2017), and induction of transcripts coding for oleosins was reported in *Spirogyra pratensis* upon heat stress (de Vries et al., 2020; de Vries & Ischebeck, 2020). During conjugation, increased levels of oleosin transcripts were found in *Spirogyra grevilleana*, suggesting their role in zygospore wall formation (Huang et al., 2013). Overall, data indicate that LDs of streptophyte algae are more similar to those found in land plants than in other Charophyta and that oleosins could already be major LD proteins in Zygnematophyceae (Dadras et al., 2022; de Vries & Ischebeck, 2020). While the process of paternal chloroplast degradation and the role of lipid metabolism in zygnematopyhcean zygospores require further investigations, the present study provides the first insights into these dynamic processes using FIB‐SEM imaging and 3D‐reconstructions.

Overall, *Zygnema vaginatum* zygospores exhibited an extremely thick and ornamented cell wall, encasing the cell with a highly protective structure. In the context of evolution and the phylogenetic position of Zygnematophyceae as the sister lineage to land plants, conjugation resulting in such protective structures was most likely crucial for the conquest of terrestrial habitats. As the harsh conditions of nonaquatic environments entail higher levels of UV‐radiation and desiccation stress, the integration of resistant compounds like aromatics in parts of their life cycle most likely gives Zygnematophycea a great advantage.

## 5 CONCLUSION

The present study provides new phylogenetic (*rbc*L) information on *Zygnema vaginatum*, coupled with a morphological species assignment. The combination of molecular analysis with classical species determination is crucial in revising and resolving the phylogenetic network within the genus *Zygnema*. Moreover, the novel application of TEM and FIB‐SEM to *Zygnema* zygospores provides ultrastructural and 3D‐analysis of *Zygnema* zygospores, their development and storage compound rearrangements. This approach enabeld the detailed investigation of the cell wall and internal cell architecture during zygospore maturation. Zygnematophyceae are the sister lineage to Embryophyta, and gaining a better understanding of their structural adaptations to land and their full life cycle is highly relevant for exploring plant terrestrialization processes. Future studies might explore the biosynthetic machineries required for producing the zygospore wall and potential similarities to spore/pollen formation in land plants, which often share a sculptured surface layering with high chemical resistance.

## AUTHOR CONTRIBUTIONS

**Charlotte Permann**: writing draft manuscript, semithin sectioning, TEM analysis, interpretation of data. **Martina Pichrtová**: collection of field samples, phylogenetic analysis. **Tereza Šoljaková**: culture isolation, light microscopy of conjugating and germinating cells. **Klaus Herburger**: TEM fixation, light microscopy of zygospores. **Pierre‐Henri Jouneau**: FIB‐SEM image generation. **Clarisse Uwizeye**: FIB‐SEM analysis and 3D‐reconstructions. **Denis Falconet**: FIB‐SEM image analyses. **Eric Marechal**: supervision of FIB‐SEM imaging and analyses. **Andreas Holzinger**: Concept and coordination, TEM analysis, manuscript writing, supervision and funding.

## FUNDING INFORMATION

This work was supported by Austrian Science Fund FWF grant P34181‐B to AH, an Early Stage Funding grant, University of Innsbruck WS717006 to CP and the Charles University Research Centre program no. 204069 to MP. PHJ, CU, DF and EM were supported by the French National Research Agency (GRAL Labex ANR‐10‐LABEX‐04, EUR CBS ANR‐17‐EURE‐0003, Alpalga ANR‐20‐CE02‐0020, DIM ANR‐21‐CE02‐0021). KH was supported by the Deutsche Forschungsgemeinschaft DFG (project no. 520755331) and an NNF Emerging Investigator grant (no. NNF21OC0067180).

## CONFLICT OF INTEREST STATEMENT

The authors declare no conflict of interest.

## Supporting information


**FILE S1.** Alignment of the sequences used to build phylogenetic tree shown in Figure 2.
**MOVIE S2.** FIB SEM generated stack of frames of a young *Zygnema vaginatum* zygospore. https://figshare.com/s/637190524e99459e5b4e.
**MOVIE S3.** FIB SEM generated stack of frames of an intermediate stage of *Zygnema vaginatum* zygospore maturation. https://figshare.com/s/30519eddaac8274263aa.
**MOVIE S4.** FIB SEM generated stack of frames of a mature *Zygnema vaginatum* zygospore. https://figshare.com/s/11bdf9f4e7ee785e7f9f.

## Data Availability

The supporting data of the present study are available upon request from the corresponding author, Andreas Holzinger. The obtained sequence of the investigated *Zygnema vaginatum* strain is available at the GenBank under the accession number OQ318179 and name 5ChO1.
